# Weighting power by preference eliminates gender differences

**DOI:** 10.1371/journal.pone.0234961

**Published:** 2020-11-05

**Authors:** Sverker Sikström, Laura Mai Stoinski, Kristina Karlsson, Lotta Stille, Johan Willander

**Affiliations:** 1 Lund University, Lund, Sweden; 2 University of Konstanz, Konstanz, Germany; 3 Stockholm University, Stockholm, Sweden; 4 University of Gävle, Gävle, Sweden; Texas A&M University, UNITED STATES

## Abstract

Power can be applied in different domains (e.g., politics, work, romantic relationships, family etc.), however, we do not always reflect on which domains we have power in and how important power in these domains is. A dominant idea is that men have more power than women. This notion may be biased because the concept of power is associated with public life. We introduce the concept of preference-weighted power (PWP), a measure of power that includes different domains in life, weighted by the domains’ subjective importance. Two studies investigated power from this perspective. In Study 1, participants generated words related to power, which were quantified/categorized by latent semantic analysis to develop a semantic measure of the power construct. In Study 2, we computed a PWP index by weighting the participants' self-rated power in different power domains with the importance of having power in that domain. Together the studies suggest that men have more perceived power in the public domain, however, this domain has a lower preference weighting than the private domain where women have more power than men. Finally, when preferences for power in different domains were considered, no gender differences were observed. These results emphasize gender difference in different domains and may change how we perceive men’s and women’s power in our society.

## Introduction

The goal of the present study was to extend the current view on power and gender by focusing on how people perceive the importance of power in different domains in their life. Whereas the current literature emphasizes power in the public domains, domains that people view as important in their life, e.g., family and friends, are often overlooked when estimating power. Here, we argue that a data-driven conceptualization of power in relation to important domains in life may enrich the current literature of power and gender. We did this by developing an empirical definition and measure that weighs the importance of different domains of power with the degree of power in that domain. Such an empirical view of power, that we call preference weighted power (PWP), provides opportunities to create a more complex understanding of the concept, which may alter to what extent we attribute power to men and women in our society. Furthermore, PWP also emphasizes, and encapsulates, that the concept of power is a subject experience, which is socially conditioned, that may change across culture, time and individuals.

Power is a multifaceted concept and there is no consensus of its definition across fields of science (e.g., [1, 2]). In a traditional sense, Townsend and colleagues [3] describe it as "a force exercised by individuals or groups" (pp.23). Other authors conceptualize power as the ability to provide and maintain resources and execute punishments [4, 5], or view power as the ability or capacity to change another person’s thoughts, feelings, or behavior so they align with one’s own desired preferences, along with the ability or capacity to resist influence attempts imposed by another person [2, 6].

However, merely focusing on general power is not enough, as power can be exerted in various ways in multiple areas of life (e.g., [2, 3, 7]). One rough distinction that has been made is between the use of structural power in public domains (e.g., politics, business, and military) and dyadic power in private domains ([8]; referring to [9]). The latter refers to the use of power to influence others in social relationships. Currently, peoples' general perception of power emphasizes public domains over power in the private domains (e.g., [8]). However, we consider that power in private domains should not be undervalued because relationships are strongly related to people’s mental and physical health, subjective well-being, pleasant affect, and life satisfaction [10–12].

Therefore, focusing only on one domain can lead to wrong conclusions about an individual's overall power. Also, research on individual preferences is completely lacking, that is, investigations of how people emphasize the subjective importance of various power domains. In this paper, we, therefore, broaden the definition of power and consider two power domains which are important in people's’ daily life (i.e., work and social relationships) as well as their subjective importance. In the present work, we aimed to further the understanding of power by studying subjectively perceived power in various domains in relation to power preferences from a gender perspective. Females are usually associated with private domains of power (e.g., family), while males with power in public domains [7], as for example work and politics.

In the public domain, power is often related to money, influential politicians, or people in executive positions at work. In these domains, men are typically overrepresented and earn higher salaries compared to women (e.g., [13–15]). Due to the strong relation of power and public domains, this might lead to the idea that men do have not only more power in public domains than women but also more power in general [16, 17]. However, private domains like family, friends, and other social relationships, which traditionally have been associated with women (e.g., [18, 19]), are often perceived as more important by people independently of gender [20, 21].

Preference weighted power. The purpose of the current work is to broaden the perception of power, by introducing a preference weighted power (PWP) perspective, where the measure of power is moved closer to how people actually view the concept compared to what is typically done using traditional scales of power. Past research has mainly focused on measuring power by using self-report measures. For example, by asking participants to indicate their encounter with power, the overall influence on other individuals, or how often they give into other people's demands (e.g., [2, 3]). We did this by developing a measure that weighs the degree of perceived power in an area with the degree of rated importance, where a domain’s ascribed high importance by people are assigned more value and are empirically weighted stronger than a domain that people perceive as less important. Here, the research question is how to weigh the importance of different domains of power to achieve a data-driven conceptualization of the power construct. PWP means that having power in a domain that is perceived as important, yields more preference weighted power than having power in a domain that is perceived as being less important. For example, a high PWP score would be generated if a person considers that his/her private life is the most important and, at the same time, have the power to make decisions in that area. In contrast, a low PWP score would be generated if an individual rate an area as important, but cannot influence others involved in the area. For example, if he/she wants, but is not allowed to influence the choice of the children's school.

Overview of the present research. Power has been defined variously, extensively studied and highlighted from different perspectives such as culture, economics, and gender (e.g., [3, 22–24]). The aim of this project is to broaden the current view of power in relation to gender, by investigating preference-weighted power (PWP). The PWP measure allows us to consider different domains where people can have power as well as to emphasize the power domains that people value as most important. By domain we refer to parts of life that are demarcated thematically (e.g., social relationships). In our investigation, we chose to explore two areas of life in respectively the public and private domains: work and social relationships. This is because work and social relationships people are two main areas in life for most people.

Two studies were conducted that used both self-rated power and statistical semantics to explore the concept of PWP. The semantic approach enabled us to empirically quantify people’s current construct of power in different domains and to generate preferences for different domains in life. In Study 1, we hypothesized that without providing a specific context, the concepts of power would be biased towards power in the work domain, as well as being associated with male power. Further, the participants would consider that the domain perceived as most important would be associated with the area where the female participants reported having power, and social relationships would be regarded as more important than work. Finally, when applying the concept of PWP in Study 2, we hypothesized that women would have more power in social relationships, while men in the work area. However, no gender differences were hypothesized in the total PWP scores, as the total PWP both take private as well as public domains into consideration.

### Latent semantic analysis

In the current paper we adopted latent semantic analysis (LSA; [25]) as a means to investigate power since LSA allows quantification and statistical analysis of verbal/written materials and enables data-driven definitions. Thus LSA provides measures of power. Words are the most common way in everyday life to measure other people's mental constructs. However, due to the difficulties of quantifying their meaning, words are an underutilized response variable. By using word clouds, we will also be able to describe and visualize the concepts of male and female powers. Previous research has not used this method to investigate power and gender. Based on the assumption that words appearing in similar contexts are associated in meaning, LSA performs a form of factor analysis called singular value decomposition on a co-occurrence matrix of a large corpus of words. These factors, or dimensions, describe how each word relates to all other words; and can, therefore, be used to statistically examine semantic aspects of words. The dimensions can be seen as coordinates in a high-dimensional space so that words that are close together within the space have a similar meaning. This semantic similarity can be represented as a numeric value (i.e., the cosine of the angle between the words) and can be used for statistical testing of meaning. LSA has successfully been applied in a variety of different settings such as analyzing the quality of essays [26], examining sensory cued recall of autobiographical memories [27], gender differences in autobiographical memory [28], detection of disordered language production as a key symptom of schizophrenia [29], and to define (e.g., [30]), measure and describe psychological constructs [31]. However, to our knowledge, LSA has not been used to analyze the perception of power and aspects of gender differences The analyses in this study were conducted in Semantic Excel (www.semanticexcel.com), an online tool for statistically analyzing semantics representation based on Latent Semantic Analysis (LSA), and developed by Sikström.

## Study 1: Measuring and describing power with statistical semantics

Study 1 applied a data-driven method to investigate how participants perceive different concepts related to power by asking them to generate keywords describing what they associate with these concepts. This enabled us to make research based empirical measures of the participants' view of the concepts, rather than apply universal definitions of the concepts from dictionaries or other authoritative references. We quantified the generated words by using Latent semantic analysis (LSA), whereby semantic similarity (SS) between the associations could be measured. Using words as a measure made it possible to visualize and quantify different power concepts with word clouds (i.e., general- male- and female power, domain of importance and sense of power in a domain). In addition, we applied a quantitative approach to measure how these concepts of different power relate to each other, and to generate important domains in life that are used in Study 2.

In this study, three hypotheses were put forward. First, we hypothesized that the participant’s associations with general power would be semantically closer to their associations of male power and work power, compared to female power and social relationship power (H1A), suggesting that the concept of general power is biased towards work power and male power. Secondly, the domain where female participants reported to have power would be semantically closer to domains related to what is important in life (H1B). Finally, words related to what’s important in life would be more associated with social relationships than work (H1C).

### Method

#### Participants

The survey (and all other surveys in this paper) was created in *Qualtrics* and afterward exported to Mechanical Turk. Data from 805 participants was collected on Mechanical Turk. Participants who did not complete the entire survey (*N* = 316) or failed the control question (*N* = 44) were excluded from the data analyses. The control question (i.e., “Answer the leftmost alternative here”) was included to omit people that did not carefully follow the instructions. The final sample consisted of 445 participants (196 male, Mean age M = 35.38, SD = 10.47; 248 female, Mean age M = 36.61, SD = 11.63; one person identified as “Other”) between the age of 18 and 67 years (*M* = 36.07, *SD* = 11.14). The majority of the sample were residents of the US (96%), while the remaining 4% resided in other countries all over the world (e.g., Australia, Canada, UK, Ireland, Germany, Spain, Sweden, India, Bangladesh, South-Korea, Jamaica, Morocco, Nigeria). Each participant was rewarded with 0.50 $ for the completion of the survey. The main rationale for recruiting participants through Mechanical Turk was that we aimed for a more heterogeneous group of participants compared to a student population (see e.g., [32, 33]). We were also able to recruit a larger sample of participants than if we had aimed for a local student population. Although there are issues associated with recruiting participants through Mechanical Turk we argue that for the current work the advantages outweigh the potential disadvantages.

#### Material and procedure

First, participants were asked to provide demographic information concerning their age, gender, and country of origin. Second, participants were asked to generate five words describing: (1) associations with general power (“Please write five keywords describing domains of power”), (2) associations with male power (“Please write five keywords describing domains where men have power”), (3) associations with female power (“Please write five keywords describing domains where women have power”), (4) associations with social relationship power (“Please write 5 words that best describe your view of social relationship power”), (5) associations with work power (“Please write 5 words that best describe your view of work power”), (6) important things in life (“Please write five keywords describing the things that are most important to you in life”) and finally (7) domains where they have power (“Please write five words describing domains in your life where you sense that you have power”).

#### Latent semantic analysis

Words are the most common way in everyday life to measure other people's mental constructs. However, due to the difficulties of quantifying their meaning, words are an underutilized response variable. Here we quantified words to study the concept of power by using Latent semantic analysis (LSA), which is both a theory and a method, to statistically measure meaning in texts. LSA allows quantification and statistical analysis of verbal/written materials and enables data-driven definitions, as well as measures of power. By using word clouds, we will also be able to describe and visualize the concepts of male and female powers. Previous research has not used this method to investigate power and gender.

### Results

#### Visualizing and describing concepts of power

**Fig 1:**
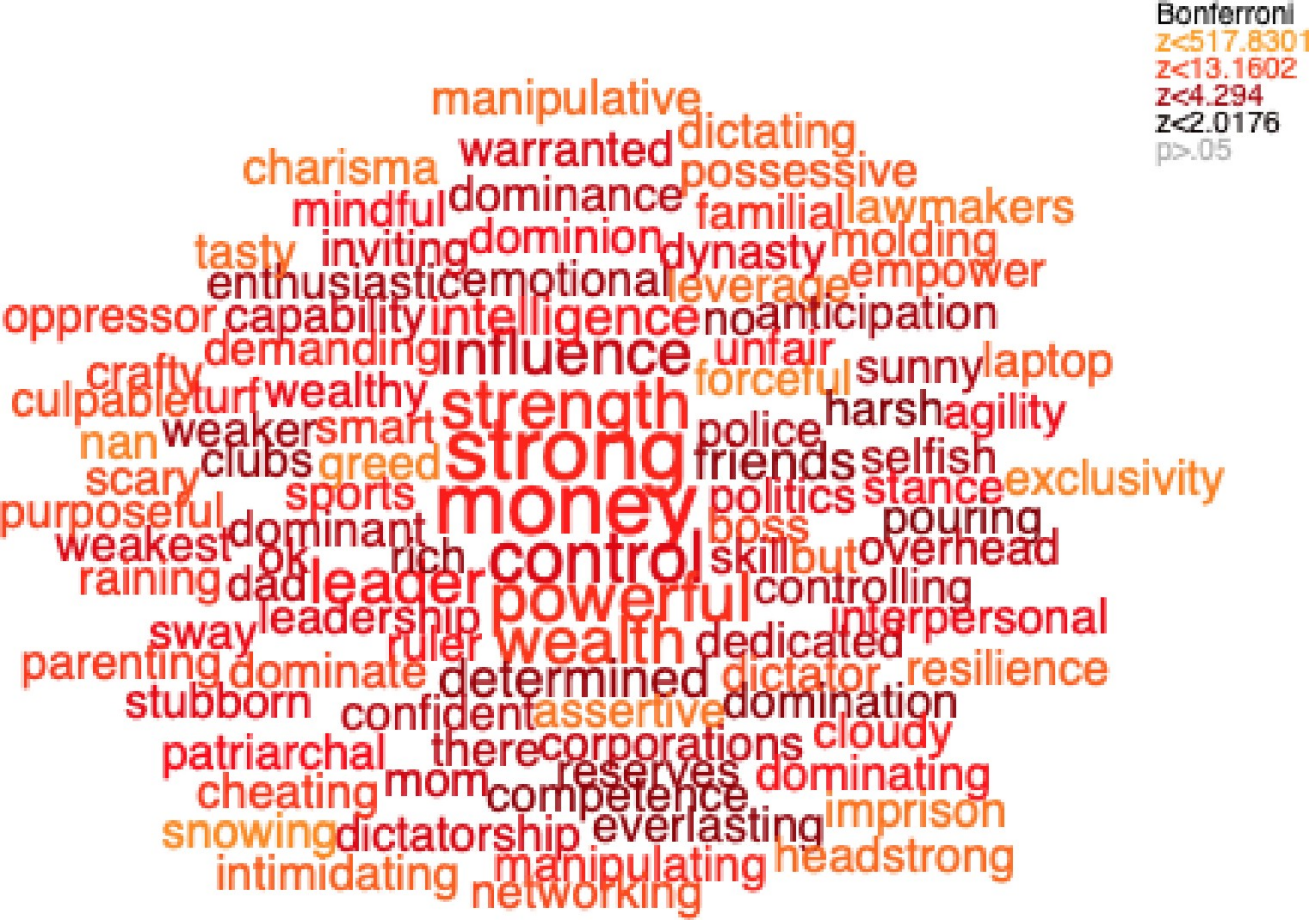
Word cloud describing general of power. *Note*. The figures were created by a semantic test (see [34] for details) comparing all word responses to the item “Write five keywords describing domains of power”(Set 1) with a random selection of equally many words sampled from the frequency distribution of Google n-grams (Set 2). The semantic test was calculated by measuring the semantic similarity (the cosine of the angle between the vectors) between a word and a vector representing the difference between Set 1 and Set 2 (where the generated words is removed). The length of all vectors is normalized to one. The semantic similarity of each uniquely generated word (Set 1) were compared to the randomly generated words (Set 2) using a t-test, and the color coding of the words indicate the z-value (see legend for coding). Words that were significant following Bonferroni correction for multiple comparisons are colored coded, and words that are significant without correction are printed in gray. Font Sizes are proportional to the square root for the frequency of occurrence of the words in the dataset. The total number of words were 2225, however the figure is limited to the 100 most significant words.

**Fig 2:**
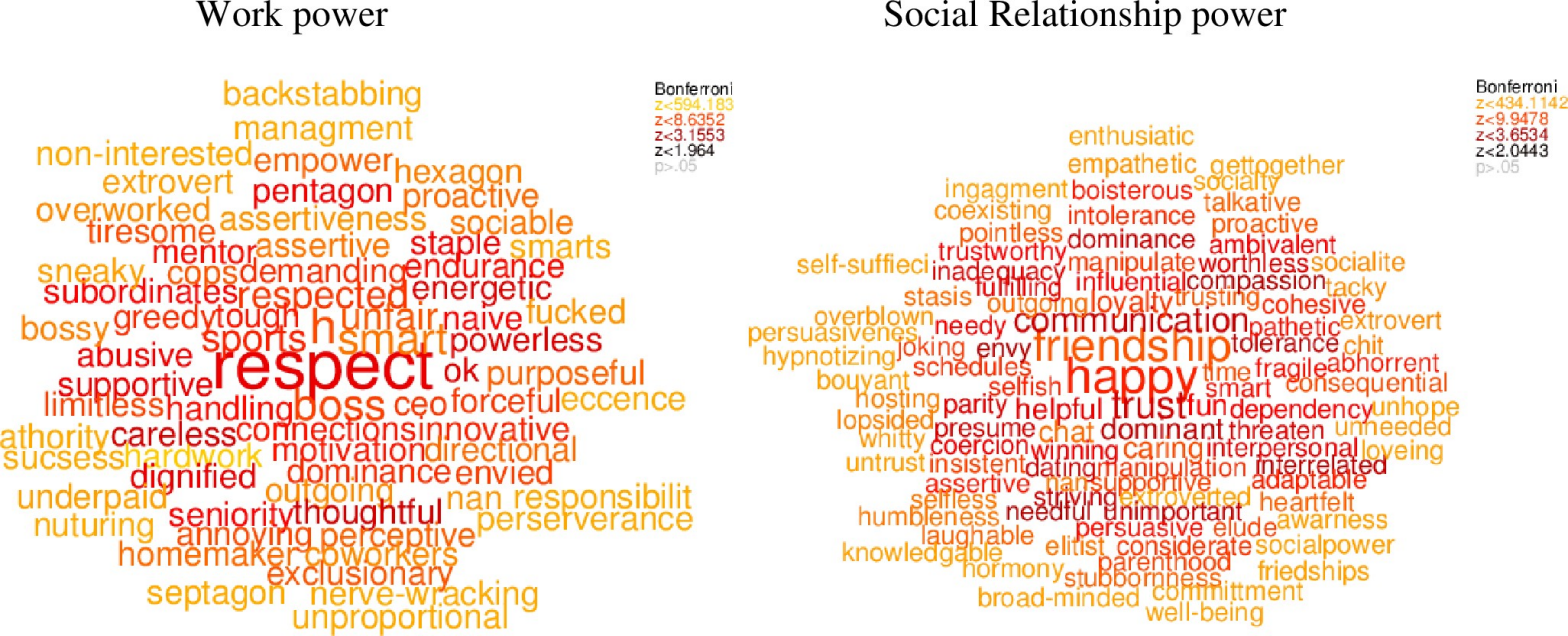
Word clouds describing the participant’s view of work and social relationship power. *Note*. The figures were created using the same method as in Fig 1, and based on word data answering the semantic questions “Please write five words that best describe your view of work power” (left panel) and “Please write five words that best describe your view of social relationship power” (right panel) were the semantic similarity were quantified in relation to a random set of words from Google N-gram.

**Fig 3:**
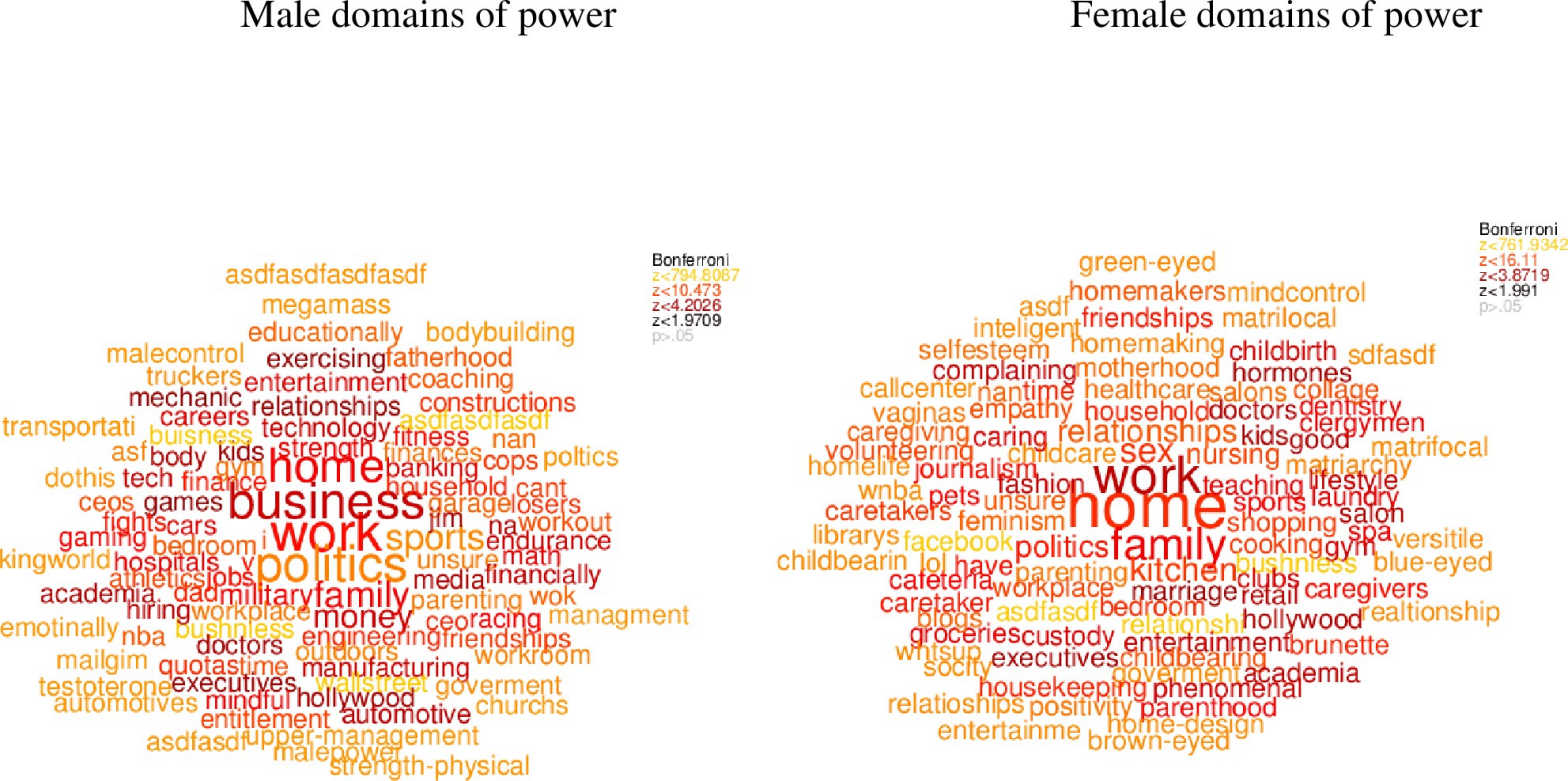
Word clouds describing domains of female and male power. *Note*. The figures were created using the same method as in Fig 1, and based on word data answering the semantic questions “Please write five keywords describing domains where men have power” (left panel) and “Please write five keywords describing domains, where female have power” (right panel) were the semantic similarity, was quantified in relation to a random set of words from Google N-gram.

**Fig 4:**
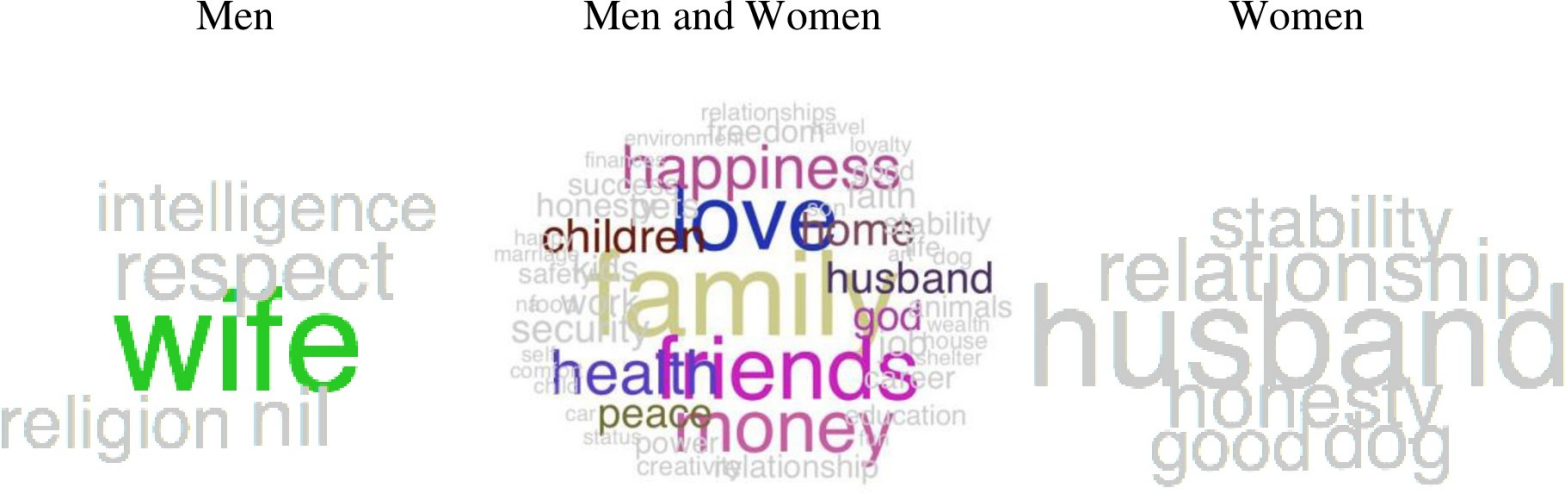
Word clouds describing what is important in life. *Note*. The women and men figure in the middle were created using the same method as in Fig 1 and based on word data answering the semantic questions “Please write five keywords describing the things that are most important to you in life”. The figure to the left also used this same method as in Fig 1, however, set 1 here consisted of words generated by women, and set 2 of words generated by men, whereas the figure to the right used the same data where set 1 and 2 were swapped. Colored words are significant following Bonferroni correction for multiple comparisons, whereas gray words are significant without corrections for multiple comparisons.

**Fig 5:**
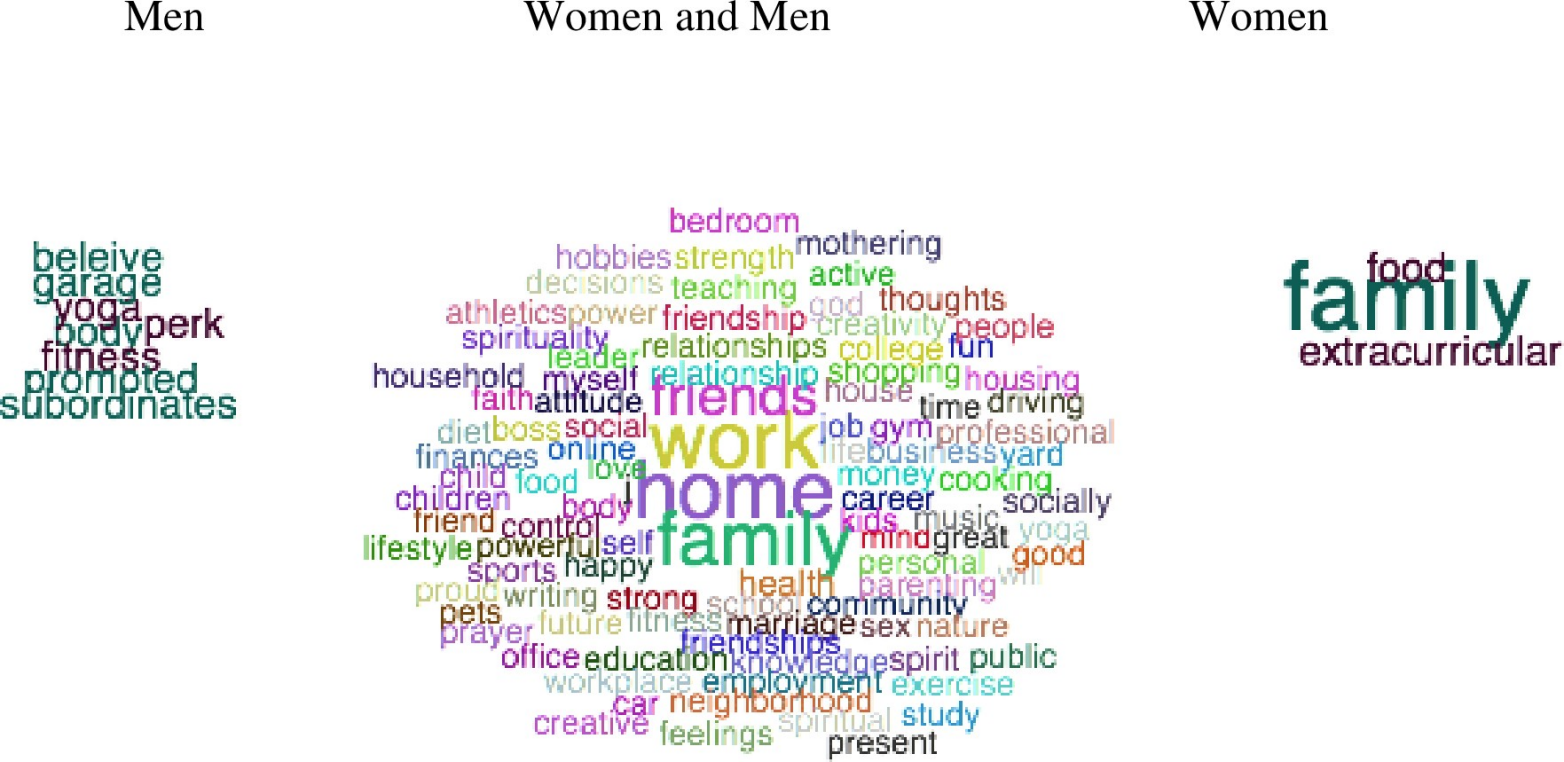
Word clouds describing domains where you sense that you have power. *Note*. The figures were created using the same method as in Fig 4, but based on word data answering the semantic questions “Please write five words describing domains in your life where you sense that you have power”.

We visualized people's concepts of general power (Fig 1), work power (Fig 2, left panel), social relationship power (Fig 2, right panel), female power (Fig 3, left panel), male power (Fig 3, right panel), what domains are important in life (Fig 4), and domains that you sense of have power in (Fig 5) using semantic word clouds (see Fig 1 footnote for methodological details).

#### Investigating general power in relation to work and male power

First, we assessed whether people’s concept of general power is biased towards their concept of work power. For this, we measured the semantic similarity between the words used to describe general power domains (Fig 1), the words the participants generated to describe domains of work power (Fig 2, left panel) and the words describing social relationship power (Fig 2, right panel). A paired t-test was conducted which showed that the words describing power, in general, were significantly semantically closer (*t*(117) = -3.51, *p* ≤ .001; Cohen’s *d* = -0.65) to the words describing work power (SS = .22) power, than to the words describing social relationship power (SS = .19). Next, we assessed whether the general power concept is biased towards people’s views of male power. To examine this, we measured the semantic similarity between the words used to describe generic power domains (Fig 1) with the words the participants generated to describe male domains of power (Fig 3, right panel) and the words describing female domains of power (Fig 3, left panel), resulting in two different semantic similarity scales. A paired t-test showed that the words describing power, in general, were significantly semantically closer (*t*(117) = 6.63, *p* ≤ .001; Cohen’s *d* = 1.23) to the words describing male power (*SS* = 0.17) power, than to the words describing social relationship power (*SS* = 0.13).

Gender differences in domains considered to be most important in life and sense to have power within. We were also interested in measuring whether the power that female and male participants have is closer related to the domains that people in general find important. This was investigated by measuring the semantic similarity between each participant answers to the questions “Please write five words describing domains in your life where you sense that you have power” (Fig 5) and “Please write five keywords describing the things that are most important to you in life” (Fig 4). By using a paired t-test, we found that words generated by women were significantly semantically closer (*t*(444) = 2.3, *p* = .0115) to words describing what is important in life (semantic similarity; *SS* = 0.43) compared to words generated by men (*SS* = 0.35).

Further, regarding what domain participants perceived as important in life, the results showed that participants used words related to social relationships more than words associated with work life (Fig 4). This finding occurs for both genders (middle panel), however, women (left panel) and men (right panel) emphasize different words. For all participants, the most frequently generated words were *family*, *love*, *friends*, *health*, *happiness*, *children*, *money*, *work*, *career* and *education* which will be further used in Study 2. These results may be compared with how participants answered the question “Please write five words describing domains in your life where you sense that you have power” (Fig 5). Here people generated words from their private life (e.g. “family”) but also words from the public life (e.g. “work”). Although many words were shared by women and men, some words showed gender differences (e.g., women emphasized power in relation to “family”).

### Discussion

Aligned with Hypothesis 1A, the words describing associations with general power were significantly semantically closer to the words describing work power, compared to the words describing social relationship power. This supports the notion that people’s concept of power is biased toward the public domain. Furthermore, the results showed a stronger power associations with domains where the participants feel men have power, compared to domains where women have power. In accordance with the hypothesis, the result suggests that participants associate power more with men than women. Importantly, the results demonstrated that the participant's concept of general power mainly focuses on work power, which is further linked to male traits power. Together, the results highlight the need of a broader definition of power, encompassing both public as well as private domains.

Furthermore, aligned with Hypothesis 1B, the words female participants used to describe their domains of power were semantically closer to words reflecting what is important in life, compared to the domain where male participants reported to have power. This indicates that women have perceived power in domains that people independently of gender perceived as important.

In sum, the results showed that people’s understanding of power is biased towards their concept of work power and associated power with domains where men traditionally have power. However, the results also demonstrated the high importance of private domains, such as social relationships. Therefore, we suggest a new measure of power in Study 2 that includes both public as well as private domains of power, weighted by their relative importance.

## Study 2: Self-rated preference weighted power

This study aimed at measuring preference weighted power. In Study 1 the participants generated words of what is important in life, and from these words, we selected the ten most commonly generated domains. For each of these domains, we asked participants three questions: how much *power* do you have in this domain, how *important* is this domain in your life, and how *important is it to have power* in this domain. The participant’s self-rated power in each domain was then multiplied with the importance to have power in that specific domain to compute the preference-weighted power (PWP) scores. We argue that this PWP is important because it removes people’s bias to overemphasize the importance of work power and broadens power to a wider set of domains that people view as important.

Gender differences in the PWP scores were investigated. When applying the concept of PWP, we hypothesized that women would have more power in social relationships, while men have more power in the work domain. However, as the overall PWP measure both includes public as well as private domains, no gender differences in the total PWP scores were expected.

### Method

#### Participants

The data of 808 participants was collected on Mechanical Turk. The rationale for using Mechanical Turk in study 2 was the same as in study 1 (see participant section study 1). Participants who did not complete the entire survey (*N* = 252) or failed the control question “Please answer alternative 3 on this question” (*N* = 18) were excluded from the data analyses. The final sample consisted of 538 participants (274 male, Mean age M = 34.13, SD = 11.31; 263 female, Mean age *M = 37*.*37*, *SD = 12*.*82;* 1 person identified as “Other”) between the age of 18 and 81 years (*M* = 35.83, *SD* = 12.36). The majority of the sample were residents of the US (83%) or India (11%), whereas the remaining 6% resided in other countries from all over the world (e.g., Germany, Italy, Macedonia, Ukraine, Singapore, Cuba, Guyana). Each participant was rewarded with 0.50 $ for the completion of the survey.

#### Materials and procedure

The survey completed by the participants was created in *LimeSurvey* (web-based software for managing surveys) and then exported to Mechanical Turk. First, participants were asked to provide demographic information concerning their age, gender, and country of origin. Second, the participants moved on to the questions related to the preference weighted power measure. From the results of Study 1, the ten most frequently generated words describing “Please write five keywords describing the things that are most important to you in life” were selected. The most important life domains related to private domains were *family*, *love*, *friends*, *health*, *happiness*, and *children* whereas the most important words related to public domains were *money*, *work*, *career*, and *education*. The participants then rated (1) how much power they have in each of these domains, (2) how important the domain is in their lives and (3) how important it is for them to have power in that specific domain (“How much power do you have in different areas of your life? By power, we refer to influence. Please choose the appropriate response for each item”; “How important are these domains in your life? Please choose the appropriate response for each item”; “How important is it for you to have power in different areas of your life? By power, we refer to influence. Please choose the appropriate response for each item”). The participants responded to the three types of questions for each of the ten domains on a 10-point Likert scale ranging from 1 (*no power/ not important at all*) to 10 (*all power/ outermost important*).

### Results

#### Factor analyses on PWP

To create a preference weighted measure of power, we multiplied the self-rated power in a domain with the importance-of-power ratings in the same domain and then aggregated this value over all ten domains. For example, if a participant rated “education” as a seven on a Likert scale measuring “importance in life” and rated his/her power in the “education” domain as a five, then multiplying these two values results in 35 as a personal PWP score for the education domain. This is repeated for all ten domains. We also calculated an aggregated PWP score by adding the results overall domains of, for example, 337.

#### Individually weighted PWP

Afterwards an exploratory factor analysis was computed on the ten PWP scores, which resulted in two components. The first component (*R*^*2*^ = .53) loaded positive to all factors and could be interpreted as a domain-independent PWP factor. This component showed no gender differences using an independent t-test. The second component (*R*^*2*^ = .13) of the factor analyses divided the PWP scores of the domains into public versus private domains, so that all factor loadings loaded in the positive direction for the items related to private domains (family, love, friends, health happiness & children), and in the negative direction for items related to public domains (money, work, career and education). This factor can thus be interpreted as a domain dependent PWP factor, which is relevant for our study. This factor further indicates that private versus public power can be seen as a continuum, where a positive value on this factor indicates higher private power, whereas a negative value suggests higher public power. This component showed a significant gender difference (*t*(535) = -3.73, *p* ≤ .001; Cohen’s *d* = -0.26), where women showed relatively higher (*M* = 0.16, *SD* = 1.04) private power than public power while men showed relatively higher public power than private power (*M* = -0.15, *SD* = 0.94). Although the largest component suggests no gender differences, the above analyses suggest a tendency that women have private power and men public power.

#### PWP separately analyzed in private and public domains

The analysis above combined data from all domains. Here we tested gender differences in private and public powers separately. Two independent t-tests were conducted, focusing either on PWP in private or public domains. First, the PWP scores (based on individual importance-of-power ratings) for all six private domains were multiplied with the corresponding factor loading of the second component that emerged in the factor analysis above. The resulting scores were then summarized to one score. Then, an independent t-test was conducted, suggesting that women (*M* = 105.90, *SD* = 40.70) have higher PWP in private domains compared to men (*M* = 98.40, *SD* = 39.91; *t*(535) = -2.16, *p* = .032; Cohen’s *d* = -0.23). This procedure was then repeated for the PWP score related to public domains, where no gender differences could be observed in regard to public power (*t*(535) = -1.24, *p* = .22).

#### Normalized PWP scores

As the PWP scores in the above section were computed by multiplying the individual importance-of-power weights with the individual's self-rated power, a participant with overall high importance-of-power ratings would obtain a higher PWP score, even if he/she does not differ from others regarding his/her self-rated power. Therefore, a lack of gender differences in PWP could be the result of an overall higher preference that is compensated by overall lower power or vice versa. To eliminate this possible artifact, we repeated the above analyses using normalized weights. The normalized weights were computed by dividing the importance-of-power rating of an individual in a domain by the sum of that participant’s importance-of power ratings for all ten domains. This way, the ten weights summed up to 1.00 and thereby solely reflect the individual relative preference for specific domains. An exploratory factor analysis was computed with the normalized PWP scores that resulted in three components. The first component (*R*^*2*^ = .24) loaded positive to all factors and showed no gender effect in PWP using an independent t-test. The second component (*R*^*2*^ = .20) of the factor analyses divided the preference weighted power scores of the domains into public versus private domains so that all factor loadings loaded in the positive direction for the items related to private domains, and in the negative direction for items related to public domains. This component showed a significant gender difference (*t*(535) = -3.64, *p* ≤ .001; Cohen’s *d* = -0.32), where women (*M* = 0.16, *SD* = 1.03) have relatively more private power than public power and vice versa for men (*M* = -0.15, *SD* = 0.94). The third component (*R*^*2*^ = 0.16) showed high factor loadings on the domains *health* (*λ* = 0.77) and *happiness* (*λ* = 0.77). An independent t-test was conducted for component three, that showed significant gender differences (*t*(535) = -2.30, *p* ≤ .022; Cohen’s *d* = -0.20), where women showed relative higher scores (*M* = 0.10, *SD* = 1.18) compared to men (*M* = -0.10, *SD* = 0.78).

#### Generic PWP scores

In the two analyses above, the PWP scores were computed using the individual importance-of-power ratings as preference weights. Here, we wanted to compare these individual preferences with generic preferences. This is important because it answers the question of whether it is people's individual preferences or people's preferences in general that account for these results. First, the generic preference weights were calculated by computing an average importance-of-power score for each of the ten domains. Then, the average importance-of-power score for a domain was multiplied with each participants’ self-rated power in the respective domain. This way, the preference weights reflected generic preferences for specific power domains, independent of gender differences in preferences. An exploratory factor analysis was computed with the generic (non-normalized) PWP scores of all ten domains, which resulted in two components. The results were very similar to the previous factor analyses. Again, the first component (*R*^*2*^ = 0.48) showed no gender differences in PWP using an independent t-test, while the second component (*R*^*2*^ = 0.13) showed a significant gender difference (*t*(535) = -2.49, *p* = .013; Cohen’s *d* = -0.22), where women(*M* = 0.11, *SD* = 1.05) had relatively higher private power than public power and vice versa for men (*M* = -0.10, *SD* = 0.94). The analyses were then repeated with generic normalized weights. Again, the first component (*R*^*2*^ = .47) showed no gender effect, whereas the second component showed a significant gender difference (*t*(535) = -2.42, *p* = .016; Cohen’s *d* = -0.21), where women (*M* = 0.11, *SD* = 1.05) had relatively higher private power than public power and vice versa for men (*M* = -0.10, *SD* = 0.10). Similar results are obtained if the generic preferences are based solely on women's preference, or solely on men’s preferences.

#### PWP based on the importance of power

Finally, we repeated the PWP analysis by using the importance of power ratings as preference weights, instead of the importance of power ratings, matched by individuals and domains. Again the results were similar to the previous analyses, with no gender differences for the first component (total PWP), while the second component dividing the data into public vs. private domains and showed significant gender difference (*t*(535) = -5.30, *p* ≤ .001; Cohen’s *d* = -0.46), where women (*M* = 0.23, *SD* = 1.04) had relatively higher private power than public power and vice versa for men (*M* = -0.22, *SD* = 0.91). The same pattern could be observed when basing the analyses on the average or normalized importance ratings.

#### Factor analyses on power, importance, and importance-of-power

The PWP analyses above were based on preference weighted power that includes both the self-rated power scores and importance-of-power/importance scores. Here we studied these scores separately, which is essential for understanding how these components contribute independently of each other. This was done by analysis, one for each of the following questions: How much power do you have in different areas of your life?; How important are these domains in your life?; How important is it for you to have power in different areas of your life? The results show a similar pattern for each question, and the components had similar interpretations as in the PWP analysis above: The first component of each of the three questions (power: *R*^*2*^ = .47; importance: *R*^*2*^ = .38; importance-of-power: *R*^*2*^ = .48) loads positive to all factors, and could be interpreted as domain independent power, domain independent importance, and domain independent importance-of-power. The first component of all three-factor analyses showed no gender effect using an independent t-test. The second component of each question (power: *R*^*2*^ = .12; importance: *R*^*2*^ = .19; importance-of-power: *R*^*2*^ = .14) divided the ten domains into public versus private domains. For the power and importance-of-power questions, items related to private domains loaded in the positive direction, while items related to public domains loaded in the negative direction. This component showed significant gender differences when using an independent t-test, associated with women having relatively more private power compared to public power (women: *M* = 0.11, *SD* = 1.05; men: *M* = -1.00, *SD* = .94; t(535) = -2.42, *p* = .016; Cohen’s *d* = 0.21) and higher importance-of-power scores for private domains than public domains and vice versa for men (women: *M* = 0.13, *SD* = 1.12; men: *M* = -0.12, *SD* = 0.85; t(535) = -2.91), *p* = .004; Cohen’s *d* = 0.25). For the importance question, the factor loadings of the second component loaded into the negative direction for private domains and in the positive direction for public domains. An independent t-test was conducted, suggesting that women value private domains more than public domains (*M* = -0.24, *SD* = 1.10) and vice versa for men (*M* = 0.23, *SD* = 0.85; t(535) = 5.67, *p* ≤ .001; Cohen’s *d* = 0.49).

### Discussion

Study 2 showed that when taking preferences into account, women have relatively more preference-weighted power (PWP) in social relationships compared to the work domain, while vice versa for men. This is aligned with earlier literature suggesting that women have more power in decision making related to private life (e.g., family, home, healthcare, daily purchases) as well as more power to influence others in relationships (e.g., [8, 35]). However, of interest, and more importantly, no gender differences were observed when investigating the domain-independent PWP factor (first factor of all PWP analyses). The results were independent of whether the weights were based on the individual or general preferences of the participants. This was still the case after controlling for possible artifacts (e.g. response biases related to overall high preferences) or when computing the PWP scores with the importance ratings instead of importance-of-power ratings. At this point it should be noted that the largest explanatory factor, related to the domain independent power factor, showed no gender differences, whereas the gender difference in the domain dependent factor accounts for a smaller effect size. In summary, the different analyses show no gender differences in overall PWP but gender differences in private and relative public power.

Furthermore, we investigated gender differences in self-rated power, the importance ratings and importance of power ratings. The results suggest that women have more private power than public power and value private domains as well as power in these domains more than public domains. In contrast, men had more self-rated power in public domains as well as higher importance and importance-of-power ratings for public domains compared to private domains.

## General discussion

As discussed in the introduction, people’s concept of power is often biased towards power in public domains (e.g, [8]), where men earn higher salaries than women and fill the majority of powerful positions in work and politics [13–15, 36]. This suggests that men hold more public power compared to women and supports the idea that men have more power in general. The goal of the present study was to broaden the current view on power and gender, by introducing the concept of preference-weighted power. Looking at people's preferences suggests that private domains, for example family, friends and romantic relationships, are viewed as being more essential in life. However, these domains have often been neglected from a power perspective. Therefore, we applied the concept of preference-weighted power (PWP). The findings from these measures converge on the idea that women have more perceived power, in social relationships which as shown in Study 1, also were the domains that were most commonly generated as the most important are in life. However, when in Study 2, weighing the relative importance of powers across domains, there were no gender differences in domain-independent power, as measured by PWP.

General power, public and male power. In Study 1 a statistical semantic approach to measure and define people's concepts of power was applied. In particular we investigated how the participants associated men and women with the concepts of power, work power and social relationship power. We hypothesized that the participants’ view of power would be biased towards their concept of work power as well as their concept of male power. Consistent with our hypothesis, the words describing the concept of work power were significantly semantically closer to the words describing power in general, compared to the words describing social relationship power. Thus, supports the notion that people’s idea of power is actually biased toward the public domain (e.g., [7]). Furthermore, the results showed a stronger association between power in general and participants' idea of male power, compared to their view of female power. That people perceive power as stereotypically masculine could imply that the participants associate men with more power than women. The data from the present study thereby confirms a socially conditioned power concept. However, the current paper also provides an opportunity to recondition these social constructs by providing empirical data suggesting that women have power in domains perceived as important independently of gender, e.g., family, friends. In addition, we explored the connection of male and female power to work versus social relationship power. The results suggest that the words created by the participants to describe their view on work power were closer related to their concept of male power compared to female power, implying that people view work power as a stereotypically masculine domain. The opposite was true for social relationship power. Here, the words generated to describe social relationship power were closest related to the participants’ view of female power, suggesting that social relationship power is perceived as a predominantly female domain of power. The results are interesting as they reflected typical gender-role stereotypes, picturing women to anticipate communal goals such as family and other social relationships, while men are expected to possess agentic traits and to pursue goals related to career and work. People often associate power with success in work related domains as well as with people who possess agentic traits. These findings are also consistent with previous literature showing that men are stereotypically perceived as more agentic, as such for example competitive, achievement oriented and assertive (e.g., [37]) and associated to executive positions at work and in politics, where people might perceive men as more powerful than women (e.g., [16, 17, 37, 38]). However, these premises might require a revision of how we look at power, as the common held view on power neglects other possible domains where power can be exerted. Thus, the result emphasizes the need to revise the concept of power in the context of gender.

The most important area in life. In study 1, we also investigated the relationship between domains where men and women have power and the domains that people think are important in life. Consistent with our hypothesis, the words female participants used to describe their domains of power were semantically closer to words describing what both genders considered important in life, compared to the words generated by men to describe their domains of power. This suggests that women have more power in domains that are important to people. As the participants primarily described private domains, when asked what is important in life, the findings are well aligned with previous literature [20, 21]. Previous research highlights the subjective as well as objective importance of private domains in life [21]. Social relationships, such as family, partner and friends are more important for people as well as their well-being and health compared to material needs, and these findings seem to be even stronger for men [10, 12, 39]. Even though in the last decades men have become increasingly involved in housework and childcare, alongside to women's larger economic and political representation and contribution [36, 40, 41], women still show a larger involvement in private domains of life (in USA; [42]) where they have power over the majority of decisions associated with family, healthcare, food, daily purchases etc. [43–45].

Preference weighted female power and power in social relationships. As addressed in the introduction, power can be defined in many different ways, and different power concepts stress different decision-making domains, depending on the specific interest of the investigation. Further, as indicated in Study 1, people often relate power with work/public power, without reflecting on other domains of power and their personal preference for these domains (e.g., [8]). Moreover, although previous research has highlighted the subjective and objective significance of private domains in life, very little prior research has been conducted in which personal preferences for these domains have been investigated (e.g., [21]). Therefore, we introduced the concept of preference weighted power in Study 2, and measured it by weighting the participants’ self-rated power with their preferences for specific domains. Consistent with our hypothesis, women had relatively more perceived power in social relationships compared to the work domains. When testing gender differences, women showed significantly higher perceived private power than men. Again, the results reflect the notion of women's larger power in private domains. This is consistent with the concept of women’s larger dyadic power, defined as the power to influence others in close relationships (e.g., [8, 35]). This study did not demonstrate men’s larger PWP power compared to women. However, men had relatively more perceived power in public compared to private domains, which is consistent with the results of Study 1, suggesting that work power is perceived as a male domain of power.

An additional focus of the study was to investigate gender differences in the domain independent PWP scores, including both private and public domains. Previous literature has often neglected private domains of power and hence attributed more power to men [16, 17, 46–48]. In contrast, no significant gender difference became apparent when using PWP as a power measure in this study and the results were consistent, regardless of the applied approach to compute the preference weights. Thus, by extending the concept of power to private domains (e.g, social relationships) and by taking preferences for specific power domains into consideration, we were able to demonstrate the possibility that men and women have similar amounts of power. This shows that, despite the still prevalent inequalities in public power, women are not powerless.

Power was investigated using common self-report measures as well as semantics. Here, including statistical semantics improved the study’s sensitivity beyond mere keyword counting. Further, we used factor analyses to assess the underlying structure of the PWP scores in Study 2. Serving as a manipulation check, the analyses supported the notion that power can be divided into public versus private power, thus enabling us to study the powers separately. In addition, applying different approaches to compute the PWP scores allowed us to control possible artefacts (e.g. response biases).

Methodological considerations. The results of the present study suggest that, despite gender differences in domain specific kinds of power, men and women do not differ in preference weighted power. However, the current project of course also has a number of limitations and shortcomings. First, as the majority of participants resided in the US, generalising the results of the study to other cultural contexts is difficult. For example, traditional gender role stereotypes, reflected by the concepts of male and female power measured in Study 1, could be even stronger pronounced in less gender-equal societies, while being less prominent in high-parity nations that are closer to achieving gender equality than the US (e.g., in the Scandinavian countries, [49]). The traditional division of labor between the genders has undergone great changes over time. As fathers perceive a growing desire to play an active part in childcare [50], the number of stay-home-fathers has increased together with an overall greater involvement in domestic tasks [51, 52]. Simultaneously, a rise in women’s economic contributions has been observed, reducing the likelihood of fathers to be the family’s main or even sole source of income [40, 41]. As men and women’s roles become more similar, people’s gender role stereotypes might change concurrently (see Social Role Theory; [53, 54]). These changes could improve gender parity, as traditional gender role stereotypes play an important part at maintaining inequalities between men and women in society [55, 56]. Additionally, to the extent that observers perceive these social roles and gender stereotypes to develop, they might also perceive power disparity between men and women to diminish (see also; [8]). Thus, prevalent gender differences in public as well as private powers might be less pronounced in future societies. However, many men still fill in the role of the major financial provider, while women adapt the role as the main caretaker in the family [42], earn less than men for comparable work and are underrepresented in high status positions at work and politics [13–15, 36]. Therefore, diminishing inequities in public domains as well as raising awareness of these problems is still of major importance.

Second, internet users are not completely representative of the general population [57, 58]. Since the studies were conducted via Mechanical Turk, the generalizability of our finding to the whole US population might be limited. As we neither included a measure of the participant’s gender role attitudes, nor investigated other potential influences like age, education or social status, the results might look different when comparing people of different age, educational and financial backgrounds. For example, previous research suggests that younger age as well as higher education and income is associated with more egalitarian or critical attitudes towards traditional gender roles [55, 59]. However, despite the generalizability problems of internet-based studies, previous research has shown that internet users are still more representative than the more commonly used convenience samples [57, 58]. Finally, the large sample sizes ensured a sufficiently high statistical power.

Finally, as noticed by the various definitions of general power, the traditional use of the concept as delineated by Townsend et al. [3] refers to different forms of power, in particular ‘power over’ and be ‘powered by’. To elaborate the matter further, power is exercised on many levels, such as between and within areas of life (e.g., [2, 3, 60, 61]). For example, a person might have high power over decisions made in their private life (e.g., parenting), while simultaneously having to obey the orders from their supervisor in a work setting. Furthermore, decision-making can also differ within domains; for example, one member of a romantic dyad could have control over the economy, and the other make decisions on how to manage specific household tasks and children's education. Thus, power is a complex construct. In the present work, forms and levels of power were not explicitly addressed. Participants freely interpreted the concept of power in general and in relation to gender, social relationships, and work. Using the same method, it would be of interest to explicitly investigate different forms and levels of perceived power.

Furthermore, semantic analyses of power could be expanded. In the present study, LSA was used to analyze power associations. LSA also enables analysis of more extensive collections of text then we collected in the present study. In a study by Townsend and colleagues [3], Mexican women’s manifestly described power experiences were analyzed with a qualitative approach. In research by Karlsson et al. [28], two quantitative methods were used to analyze women's and men's memory reports. LSA to measure the latently described (i.e., the underlying meaning in the expressed words) and linguistic inquiry word count (LIWC; e.g. [62]) to measure the manifestly described (i.e., the actual words said). In line with the present work, it was found that the female participants latently were oriented towards social relationships in their memory descriptions than the male participants were. Thus, there are many analytic methodologies that could shed further light on the dimensions of power.

In sum, the results illustrate how people’s definition of power is biased towards public domains, which are further stronger associated with men than women. However, it was also found that women have more perceived power in the private domain. This highlights the need to broaden our perception of power, as power can be exerted in many important domains in life. Because when considering many different domains of power, weighted by their relative importance (PWP), we demonstrated a lack of domain-independent gender differences in preference weighted power. Taken together, the results of this project may significantly change how we perceive power.

## Supporting information

S1 Material (ZIP)
