# Supplementary material for: Weighting power by preference eliminates gender differences
Source: PLoS One. 2020 Nov 5;15(11):e0234961. doi: 10.1371/journal.pone.0234961 (PMC7644059; doi:10.1371/journal.pone.0234961)
Supplement: S1 Material — (ZIP) [file pone.0234961.s001.zip › Data_PLOS_ONE/Manuscript_Data_and_Variablenames.docx]

**Study 1: Semantic Affect Measure**

- Anonymous manuscript version: **Study1_Workingdata 3 reduced Semantic Affect Measure, datainsamling 2.sav**

**Study 2: Workingdata Norms & Power 2**

- All PWP versions: **All_PWPs_Study2_Workingdata Norms and Power2_2.sav** Anonymous Only manuscript Versions: **Study2_Workingdata Norms and Power2_2.sav**

| Analysis Manuscript | Variable Name SPSS |
| --- | --- |
| Individually weighted PWP | PW_family  PW_friends  …. |
| PWP separately analyzed in private and public domains: | PW_SRDomains_Manual_FactorAnalysis  PW_WorkDomains_Manual_FactorAnalysis |
| Normalized PWP scores: PW9 | PW9_family  …. |
| Generic PWP scores | PW3_family  …. |
| PW weighted by importance of domain | PW2_family  … |
| Factor analyses   - 1. Self-rated-power:   2. Importance of domain:   3. Importance of power in domain: | 1. FAC1_/FAC2_Power 2. FAC1_/FAC2_ImportanceDomain 3. FAC1_/FAC2_ImportancePower |
